# The role of a salt pillow in deep saline aquifer integrity and shallow groundwater resources

**DOI:** 10.1038/s41598-025-99721-2

**Published:** 2025-04-29

**Authors:** Jolanta Putnaite, Alireza Malehmir, Morten Bjerager, Tanni Abramovitz, Henrik Vosgerau, Marie Keiding

**Affiliations:** 1 Department of Earth Sciences, Uppsala University, Uppsala, 75236 Sweden; 2 Geological survey of Denmark and Greenland, Copenhagen, 1350 Denmark

**Keywords:** Salt pillow, Deep saline aquifer, Gassum Formation, Onshore seismic acquisition, Landstreamer, Seismology, Tectonics, Geology, Hydrogeology, Carbon capture and storage

## Abstract

Salt pillow growth leads to the deformation of the suprasalt strata and can affect geological carbon storage in saline aquifers. A concern for storage sites on land is the drinking water because the impact of the deformation on fluid pathways and groundwater systems needs to be studied. Here, we present new seismic data from a potential carbon storage site in Denmark. A high-resolution seismic dataset was acquired using a strategically deployed nodal array and a landstreamer to assess the effects of the Permian Zechstein salt migration on the layers above a salt pillow. We create a geological model of the subsurface through correlation of reflection data with borehole data and investigate the main Upper Triassic Gassum Formation reservoir. Our findings reveal a fault system linked to salt diapirism and evidence of salt pillow growth from the Triassic into the Miocene. We image up to 2 km-wide Quaternary palaeovalleys, which incise the deformed overburden and serve as key groundwater aquifers. These results have implications for fluid transport in deep saline aquifers above the salt pillow, and for clarifying the deformation impact on the shallower groundwater systems.

## Introduction

Salt tectonics^1,2^ is a key research topic that is relevant to the origin and evolution of saline aquifers as potential geological carbon storage (GCS) sites in many sedimentary basins. Rock salt is weaker and more prone to movement than most other rocks. It migrates in response to gravitational forces or lateral forces of regional tectonics^3^. These mechanisms drive the salt flow and can generate salt pillows^4^. In contrast to diapirs, salt pillows have concordant contact with the overlying strata. The overburden is gently folded, often forming broad structural closures. This is significant for the capacity and connectivity of saline aquifers in the overburden, influencing reservoir distribution, fluid flow and integrity. Despite efforts to study these structures, detailed investigations are limited due to restricted access or insufficient resolution of subsurface geophysical data.

**Fig. 1:**
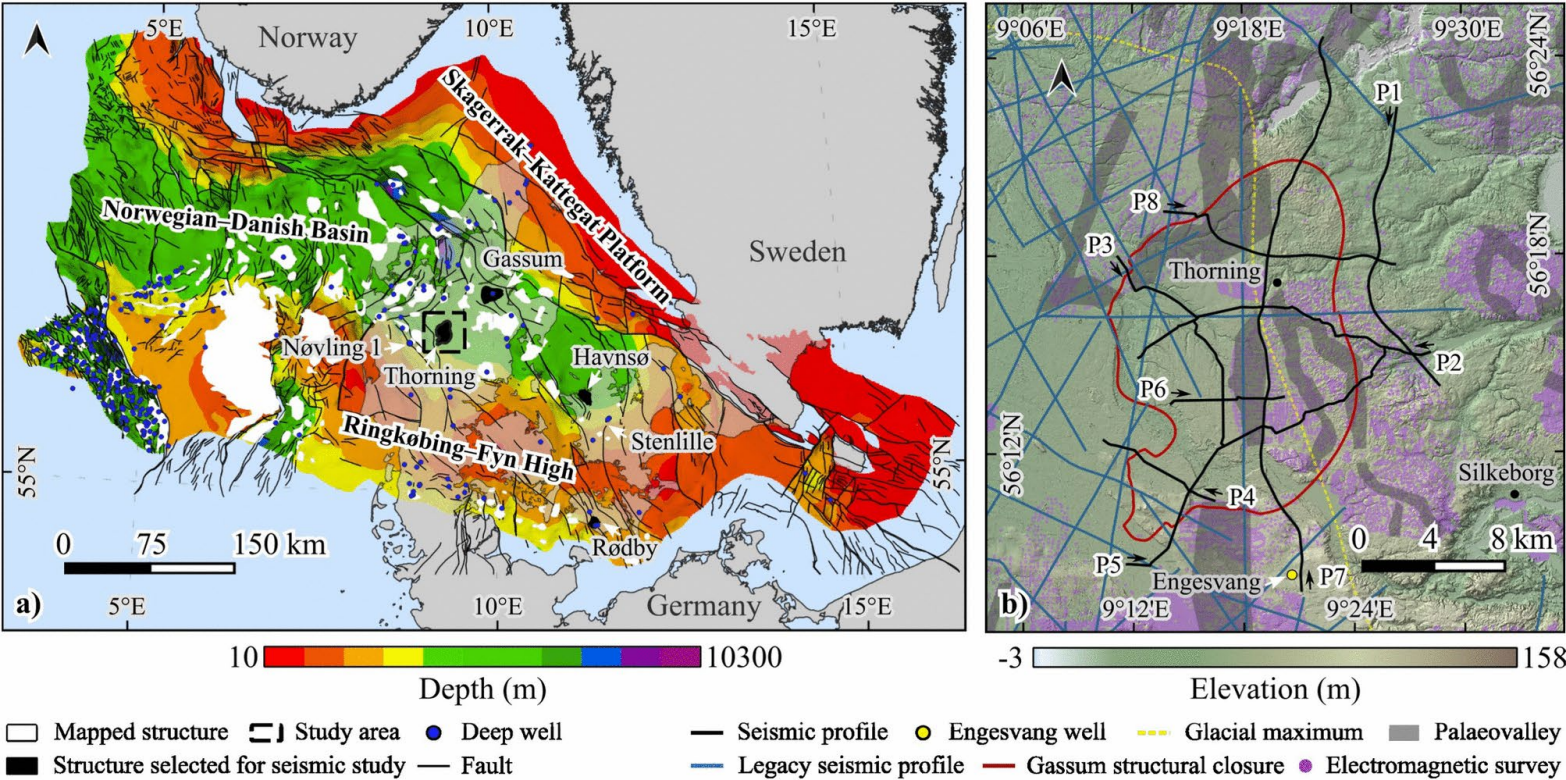
Location and structure of the Thorning seismic survey area. (**a**) Depth map to the top pre-Zechstein in the Norwegian–Danish Basin^20^ showing cross-cutting faults, deep wells and mapped structures with potential for geological carbon storage (GCS). The specified structures (Stenlille, Havnsø, Gassum, Rødby, and Thorning) were investigated as part of the seismic campaign for GCS conducted during 2022–2023. The study area of the Thorning salt pillow is marked inside the black rectangle. The closest deep well is Nøvling 1. (**b**) The high-resolution LiDAR terrain data showing Thorning survey layout (profiles, P1 to P8) along with prior electromagnetic surveys^12^ and the Engesvang shallow well location. Black arrows show the seismic acquisition direction. The structural closure of the Gassum horizon at 1850 m (below sea level) depth based on legacy seismic data^5^ is marked with a red contour line. Maps were made using QGIS 3.22 [https://www.qgis.org/] and underlying data were extracted from open data sources [https://eng.geus.dk/products-services-facilities/data-and-maps/subsurface-data-denmark; https://eng.geus.dk/products-services-facilities/data-and-maps/maps-of-denmark;https://dataforsyningen.dk/data/3931].

Following the 2020 evaluation of GCS potential in Denmark^5^, a series of seismic studies were conducted with the focus on five onshore areas related to salt pillows. Figure 1a shows the interpreted reservoir closures for the Danish part^5^ of the Norwegian–Danish Basin^6^, indicating selected seismic investigation sites. The seismic surveys aimed to assess different structures for GCS while considering environmental factors. Groundwater is the primary water supply in Denmark and has been mapped using electromagnetic methods throughout the country^7–12^. It is known that groundwater is present in porous sedimentary media, which comprises Quaternary sand and gravel, Miocene sand and gravel, and chalk^10^. Given the significance for drinking water, their interrelation with salt tectonics have not been investigated in detail, because this has not been possible with the legacy seismic data (often poor quality and sparse) in Denmark.

In 2022, a pilot survey was conducted to support the effort of characterizing potential GCS sites in Denmark with an experimental work in the Stenlille gas storage area^13,14^. The operational site has a wealth of information that helped to assess the performance of the pilot study. A novel onshore seismic data acquisition solution combining a micro-electromechanical sensor (MEMS) based landstreamer and a nodal array^15,16^ was tailored explicitly for cost-effective, high-resolution, full-depth range imaging purposes. The success of the Stenlille pilot survey^13^ led to one of the most extensive recent onshore seismic campaigns $(>650 \text{ km }$ of seismic profiles) for GCS over five salt pillow sites (Stenlille, Havnsø, Gassum, Rødby and Thorning) in Denmark^13,17–19^ (Fig. 1a) and set grounds for further up-scaling and development work. These new seismic surveys showcase a unique opportunity of applying environmentally friendly, cost-effective acquisition, and imaging strategies onshore while collecting high-quality open-access data in urban environments.

Here, we focus for the first time on the seismic survey at the Thorning salt pillow site (Fig. 1), conducted between August and October 2023. We present the results of the combined deep and shallow high-resolution seismic acquisition solution to simultaneously image the salt pillow and suprasalt strata, including the overlying shallower groundwater systems. The unique feature of the study area is the abundance of Quaternary palaeovalleys, which can provide insight into the interrelation between the palaeovalleys and deformed overburden. The new results are critical not only for investigating the Upper Triassic Gassum Formation for $\hbox {CO}_2$ storage and mapping groundwater resources but also help to recognise the importance of detecting faults between the candidate $\hbox {CO}_{2}$ reservoir and drinking water aquifers.

## Results

### Seismic data analysis

**Fig. 2:**
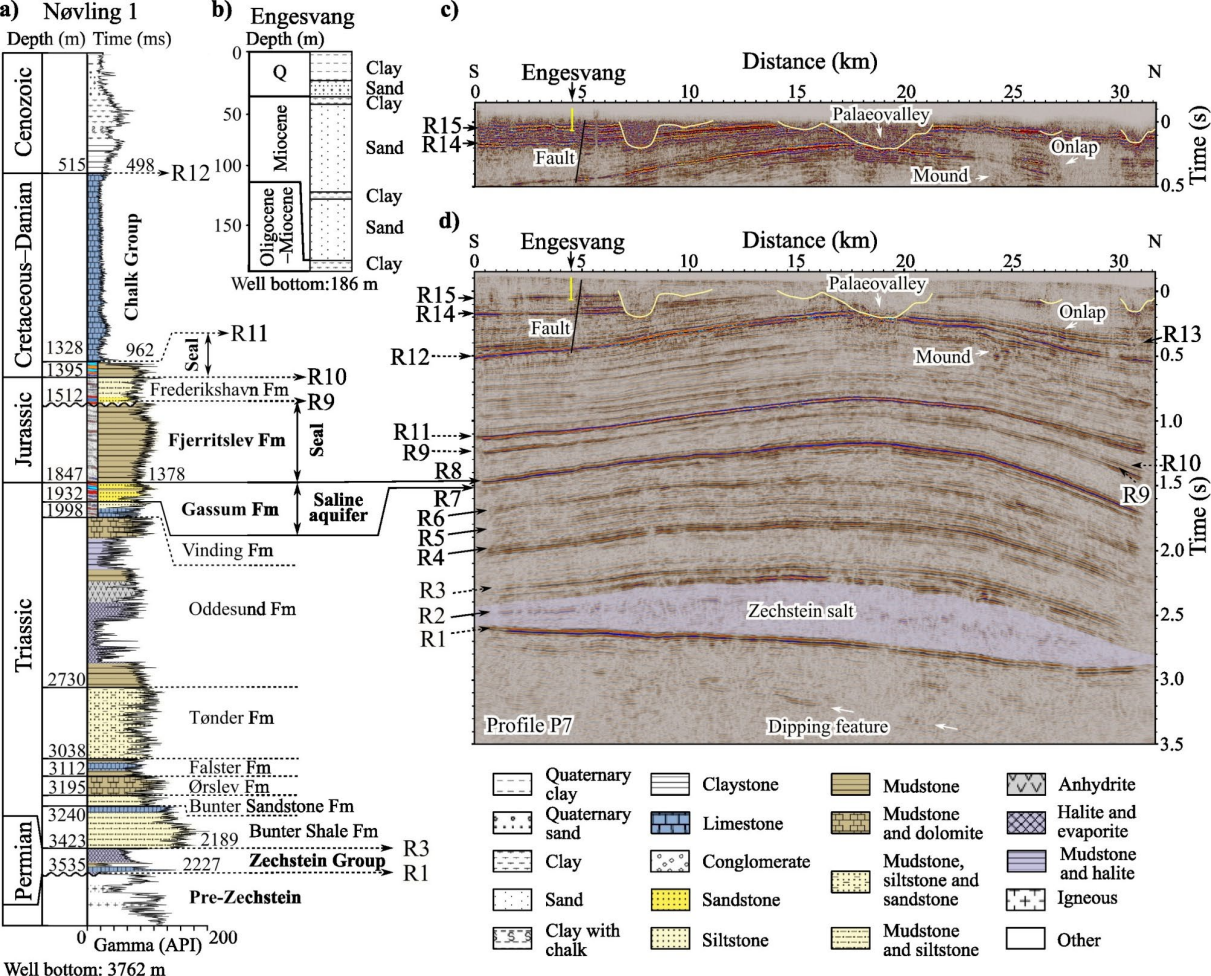
Correlation of the lithostratigraphic units with the primary seismic features. (**a**) Lithology and stratigraphy are shown from the deep Nøvling 1 well^21^, superimposed on a gamma-ray log and supplemented with time data from a sonic log. The lithology patterns and colours are defined in the legend. Unconformities are indicated by wavy lines. An example of seismic polarities is provided for the top Oddesund Fm-base Chalk Group interval. (**b**) Shallow Engesvang well data. (**c**) A near-surface segment of the seismic section P7 traversing the Thorning salt pillow from the broadband landstreamer dataset, (**d**) alongside section P7 from the nodal data. The primary seismic features (R) are marked as R1–R15. The Nøvling 1 well is at 30–40 km distance from P7 and the corresponding lithostratigraphic units are traced through the legacy seismic profiles between the well and the seismic section. Yellow vertical line at the top of the seismic sections indicates the location of the Engesvang well. Figures were made using SKUA-GOCAD22 [https://www.aspentech.com/en/products/sse/aspen-skua] and Inkscape 1.3.2 [https://inkscape.org/], while the well data were extracted from open data sources [https://data.geus.dk/JupiterWWW/].

Figure 2 shows the relationship between lithostratigraphy^21^ and one of the seismic sections. We combine data from the closest deep Nøvling 1 well (Figs. 1a, 2a), the shallow Engesvang well (Figs. 1b, 2b), and the south–north trending longest (33 km) seismic profile P7 (Fig. 1b) for the landstreamer (Fig. 2c) and nodal (Fig. 2d) data. The deep well is located 30–40 km southwest of P7 towards the Ringkøbing–Fyn High, close to the Nøvling salt diapir^22^. The geometry of seismic features between R1 and R3 suggests that the Zechstein salt is concordant to the overlying strata and forms the Thorning salt pillow. Furthermore, the salt pillow rests on a tilted pre-Zechstein strata having an angular unconformity at the base Zechstein Group (R1) where different lithological units subcrop. This is supported by the observed dipping features below R1 in the seismic sections and the unconformity and thin intervals of variable pre-Zechstein rocks described in the Nøvling 1 well data. The relatively thin Zechstein Group in the well consists of mudstone, dolomite and limestone layers embedded between anhydrite beds with pure salt intervals. The high-amplitude feature, referred to as R2, within the salt pillow is laterally discontinuous. The characteristic internal diffractivity (Supplementary Figure S1) may originate from discontinuities within the layered sequence of the Zechstein Group. The discontinuities form due to salt movement and can diffract the seismic energy. Interestingly, R3–R15, which are associated with the Triassic–Miocene suprasalt strata, are gently folded.

The saline aquifers below the Chalk Group, where the depth is greater than 1 kilometre, are potential geological reservoirs for carbon storage (Fig. 2), as carbon dioxide can remain in a supercritical state. A seismic polarity reversal is observed at R11 (base Chalk Group) where the minimum phase wavelet starts with a positive amplitude (blue or white) instead of the negative amplitude (red or black) seen in the first arrivals, as shown in Fig. 2. The polarity reversal is due to the lower acoustic impedance layer below the Chalk Group, confirmed by the sonic log data and velocity model building. As a result, the correlation of the reservoir and seal formations with the corresponding seismic features is more straightforward. The main reservoir consists of the Upper Triassic sandstone of the Gassum Formation, bounded at the top by a characteristic high-amplitude seismic feature, R8, and at the base by low-amplitude seismic feature, R7, with an average thickness of 100 m between them. It is overlain by a seal composed of the Lower Jurassic mudstone of the Fjerritslev Formation, which continues for approximately 200–500 m up to R9. The thickness of the Jurassic interval (R8–R10) is treated with caution due to the presence of a basin-wide Jurassic unconformity^23^ (Fig. 2a). A potentially porous interval of the Frederikshavn Formation, above R9, is only half the thickness of the sandstone of the Gassum Formation based on the data from the Nøvling 1 well, but it is still effectively imaged. The overlaying seal consists of the Jurassic–Lower Cretaceous mudstone below R11. Furthermore, well data suggest the presence of additional formations, which may contain porous rocks at depths of 2–4 km below the Oddesund Formation that need further petrological analysis.

**Fig. 3:**
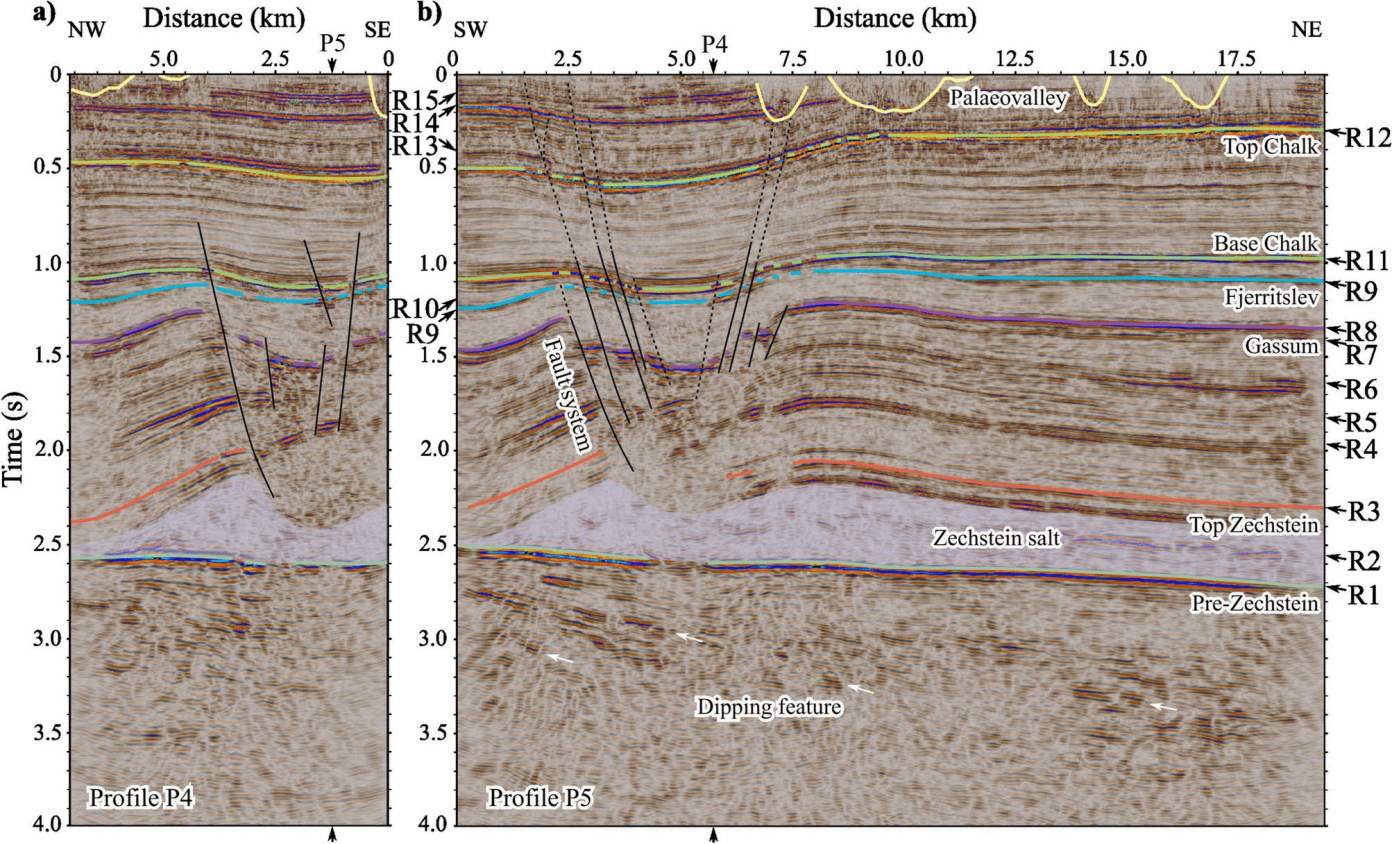
Orthogonal seismic sections showing the fault system. Nodal seismic data of (**a**) P4 and (**b**) P5. Seismic horizons were picked along the primary seismic features, which are correlated with the borehole data, and named after the associated lithostratigraphic units. Locations where the profiles intersect are indicated with arrows. Figures were made using SKUA-GOCAD22 [https://www.aspentech.com/en/products/sse/aspen-skua] and Inkscape 1.3.2 [https://inkscape.org/].

The fault system shown in Fig. 3 cuts through the Gassum horizon in the southwestern part of the study site. It is defined by sets of conjugate normal faults that form a graben. Fault displacement is the largest within the Triassic succession below the Gassum horizon (Fig. 3) reaching $>450$ m throw. Bended seismic features are observed against the faults that form fault-related folds, which further influence the geometry of the horizons. A Jurassic succession below the Fjerritslev seal that is thicker on the hanging wall than on the footwall indicates syn-sedimentary (growth) normal faulting. Although the Jurassic succession above the Fjerritslev horizon is less affected by fault displacement, its thickness was controlled by the underlying palaeo-topography, resulting in thinning over the highs and thickening over the lows. The fault patterns can be traced into the Chalk Group and the unconsolidated Cenozoic layers up to the top of the seismic section (Fig. 3). However, the connectivity of the faults up through the sections is less clear. Fractures and faults cutting through the Gassum horizon with throws up to approximately 50 metres are also imaged above the crest of the Thorning salt pillow in the seismic sections P2, P3, P6, and P8, of which the largest occur along P2 presented in Supplementary Figure S2.

**Fig. 4:**
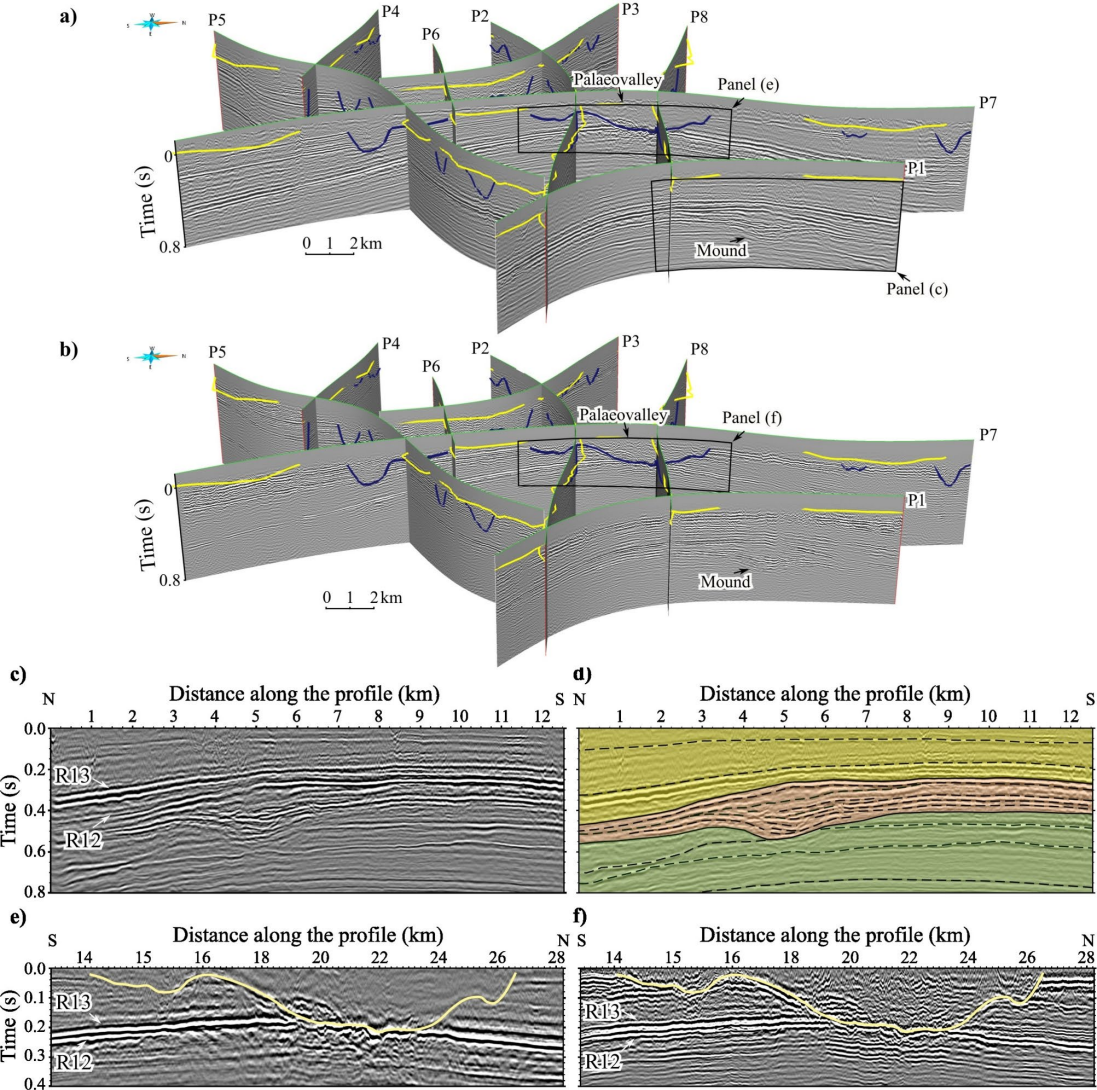
Near-surface features of the nodal and landstreamer seismic datasets. Three-dimensional (3D) view of the survey layout (Fig. 1b) showing seismic sections from the (**a**) nodal and (**b**) landstreamer datasets. The yellow lines represent the seismic profile locations measured at every seismic recorder position along the roads. (**c**) A segment from the nodal data along P1, where mound-shaped features were imaged in the northern part of the survey area. (**d**) Interpretation of the corresponding segment with different colours distinguishing the mound-shaped interval (orange) from the layers above the top Chalk horizon (yellow) and below the mound (green). The dashed lines mark the seismic image patterns. A segment from the (**e**) nodal and (**f**) landstreamer datasets along P7 showing an example of the palaeovalleys imaged with the two different acquisition systems. Locations of the mounds and palaeovalleys displayed in the panels are indicated with black arrows in (**a**, **b**). Figures were made using SKUA-GOCAD22 [https://www.aspentech.com/en/products/sse/aspen-skua] and Inkscape 1.3.2 [https://inkscape.org/].

Figure 4 focuses on shallower portion of the nodal and landstreamer datasets. Distinctive features are observed along P1 and P7 towards the northern edge of the survey area shown in Fig. 4a–c. They are characterized as mound-shaped, semi-continuous to discontinuous seismic features. These features are present below the top Chalk horizon (R12), where a Danian succession (Palaeocene) is expected based on the well data (Fig. 2a). The occurrence of Upper Cretaceous-Danian mounds and channels is known from previous studies of shallow seismic profiles and outcrops in the Norwegian–Danish Basin^24–26^. Figure 4d shows our interpretation of the mounds along P1. Our observations help us to ensure that we understand the structure of the upper Chalk Group where these mounds occur.

The Quaternary palaeovalleys identified in the study area range in width from a few hundred metres to several kilometres. Compared with the nodal sections (Fig. 4a), the broadband landstreamer sections (Fig. 4b) show stronger amplitudes and higher seismic resolution, resulting in a more detailed image of the palaeovalleys. The palaeovalleys are carved into the Palaeogene-Neogene succession by glacial and glacial meltwater^27^ down to approximately 0.25 s. The deepest palaeovalleys incise into the top Chalk horizon (R12), as seen in Fig. 4e–f. The valley bottom topography shows an undulating pattern and is dominated by a U-shaped morphology. Because there are no available well data to correlate with the complete infill of the channel, this interpretation is based solely on the seismic data and shallow wells such as the Engesvang well (Figs. 1b, 2b). The valley infill patterns, which show diffractivity (Supplementary Figure S3), suggest the presence of boulders and cobbles. The channel incisions indicate that there may be several phases of valley formation and sediment accumulation along the channel systems (Fig. 4e–f).

### 3D structural configuration

**Fig. 5:**
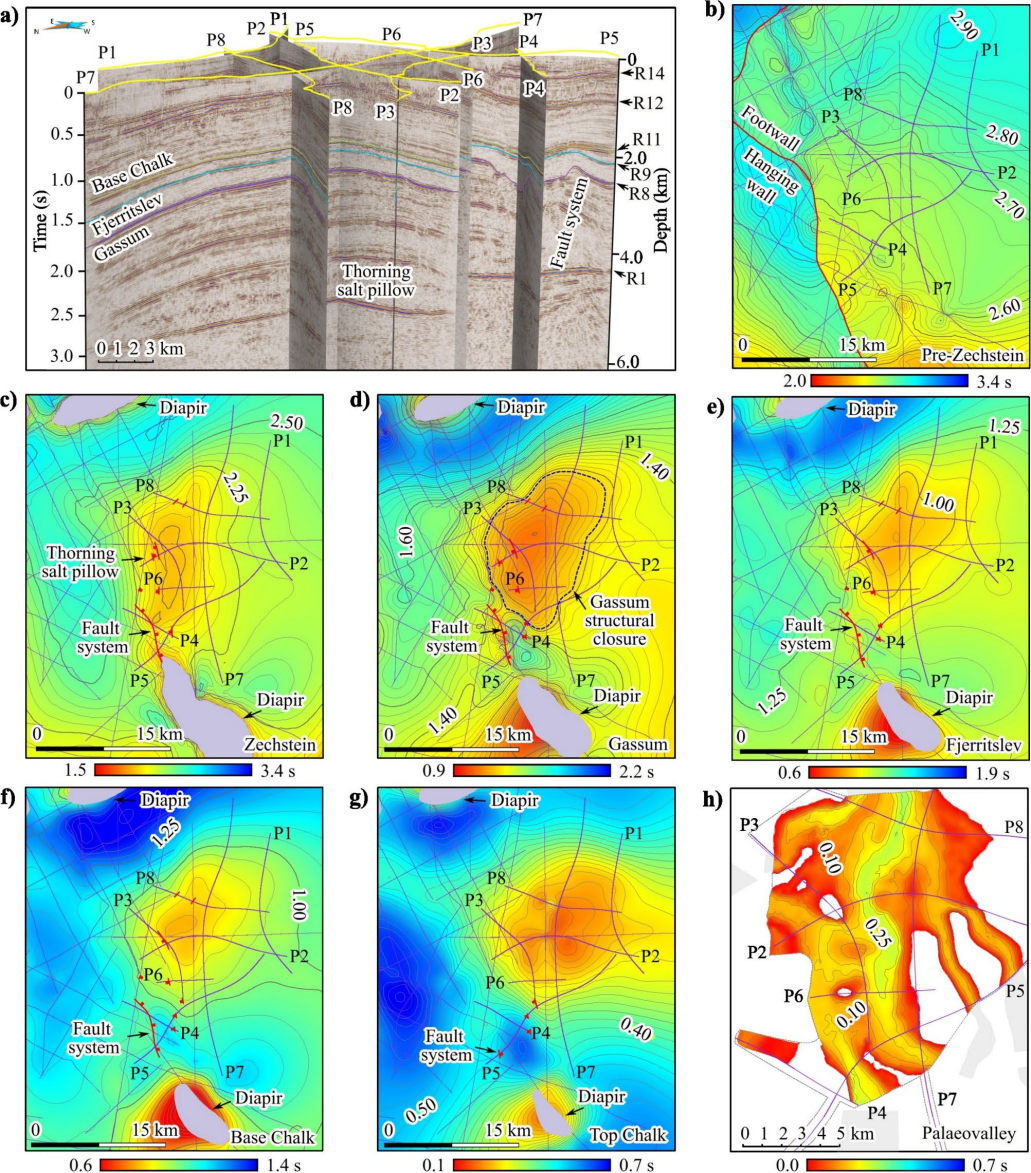
3D model of the Thorning site. (**a**) 3D view of the new seismic sections of the nodal data. The black arrows point to some of the primary seismic features from Fig. 2. Structural time maps: (**b**) top pre-Zechstein, (**c**) top Zechstein, (**d**) top Gassum, (**e**) top Fjerritslev, (**f**) base Chalk, (**g**) top Chalk and (**h**) palaeovalleys that incise the pre-Quaternary layers. The purple lines show legacy and new seismic profiles used to create the structural time maps. The red lines mark faults, the purple areas map the salt diapirs, and the dashed blue contour line indicates the top Gassum structural closure (1.28 s). The grey areas represent the palaeovalley map (Fig. 1b) and the dashed grey contour line show model boundaries. Figures were made using SKUA-GOCAD22 [https://www.aspentech.com/en/products/sse/aspen-skua] and Inkscape 1.3.2 [https://inkscape.org/].

The three-dimensional (3D) geometry of the subsurface was investigated by mapping the primary seismic features in all available seismic sections, including the legacy seismic profiles (Fig. 1b), as shown in Fig. 5. Reflectivity and seismic imaging are poor within the salt bodies, and the seismic profiles have irregular two-dimensional (2D) seismic coverage. However, the extent of salt diapirism and successive faulting are still well-imaged.

The pre-Zechstein horizon (Fig. 5b) reveals a high-angle SSE–NNW trending fault ($>50 \text{ km }$ long). It does not appear to transect the top Zechstein in the study area (Fig. 5c). Subparallel to the fault strike on the tilted footwall block (Fig. 5b) is the Thorning salt pillow (Fig. 5c). An elongated SSE–NNW oriented salt diapir is present along the fault strike southward from the salt pillow over the footwall block (Fig. 5c–g). In contrast, the salt diapir, which is located further north along the fault trace, is directly above the fault (Fig. 5b–g). The distribution of the Zechstein salt (Fig. 5c) suggests a relationship with the top pre-Zechstein and the transecting faults (Fig. 5b).

The interpreted structural closure of the main Gassum reservoir is truncated by the fault system at the SW edge (Fig. 5d). The fault system separates the Thorning salt pillow and the elongated salt diapir south of it (Fig. 5c–g). Faulting is the most significant along seismic sections P4–P5 and becomes less evident towards the northern edge of the Thorning salt pillow (Fig. 5d). The geometry of the crooked acquisition line combined with the Quaternary palaeovalley network complicates the identification of the fault system. Still, the use of the dual-element acquisition system that combines landstreamer and nodal array provides an advantage of two independent datasets, contributing to an improved fault detection. Faults are more clearly expressed in the broadband landstreamer dataset compared with the nodal data for the shallower part of the seismic sections (Supplementary Figure S3). Furthermore, diffractivity, which is observed in the unmigrated seismic sections (Supplementary Figure S3), strengthens fault interpretation. Edge diffraction is associated with sharp discontinuities in stratigraphy that are generated by faults. Therefore, several faults can be traced up-dip through the seal and into the Chalk Group succession, as shown in Fig. 5c–g.

Figure 5h shows the palaeovalley system of Quaternary age extending above the Thorning salt pillow. The channels were constructed manually by tracing valley margins in the new seismic sections, which have sufficient resolution near the surface compared with the legacy seismic profiles, and integrating them with the palaeovalley map (Fig. 1b). The palaeovalley map in Fig. 1b is primarily based on previous transient electromagnetic data^12^ collected for the mapping of palaeovalleys due to their potential to act as groundwater aquifers^12^. The deepest valley incisions have a preferred N–S orientation affecting the top Chalk, as seen in Fig. 5g–h. There is no clear relationship between the shape of the channels and the geometry of the top Chalk at the crest of the Thorning salt pillow. However, the southernmost channels follow the fault system imaged in P4–P5 (Fig. 3), where they traverse through normal faults, and continue above the salt diapir following a similar SSE–NNW trend (Figs. 1b, 5c–h). Notably, the direction and distribution of the Quaternary palaeovalleys is also consistent with the glacial maximum (Fig. 1b). The cross-cutting relationships between the N–S oriented channels and roughly E–W geometries indicates the presence of multiple generations of valley formation.

The topography of the study area has a transient morphology that can be divided into the eastern half of glacial complexes with sharp palaeoriver valleys cutting through moraine highlands and the western half dominated by more subtle proglacial meltwater deposits (Supplementary Figure S4). The palaeoriver network has a prominent valley pattern (Supplementary Figure S4) that closely aligns with the geometry of the top Chalk (Fig. 5g). Here, the lower-lying terrain along the edge of the deformed suprasalt strata, which forms a dome, may have guided the course of the channels.

## Discussion

The proposed triggering mechanisms for salt tectonics in the Norwegian–Danish Basin include faulting and tilting of the sedimentary basin floor, differential loading, halokinesis, where salt moves due to gravitational differential loading^6,28,29^, and regional tectonics^30,31^. The 3D subsurface model constrained by the seismic data (Fig. 5, Supplementary Figure S5) suggests that the top pre-Zechstein and transecting faults play an essential role in the formation and evolution of the salt bodies and their subsurface geometry. This is evident from the imaging of the Zechstein salt base, which illustrates the relationship between the diapir and the salt pillow, as well as the underlying structures. The salt pillow formed along the fault strike between the salt diapirs (Fig. 5b,c). The salt swell declines laterally to thinner salt or welded salt (Supplementary Figure S5), while, as shown along P4 and P5, it is truncated by the fault system. These observations suggest that the salt pillow and the southern salt diapir may have formed a more singular structure during the early stages of their evolution. This relationship may have been influenced by the location of the salt diapir, which is in the highest structural area.

The Triassic succession above the crest of the salt pillow is more folded compared with the overlying layers (Fig. 5, Supplementary Figure S2), which could be evidence for the onset of salt movement. This is supported by the ideal 2D representations of salt tectonics (Supplementary Figure S5). The fault throw is the largest at the Triassic Gassum interval, and the thickness variations of the Jurassic interval along the fault system suggest syn-sedimentary faulting. These observations are relevant for the carbon storage potential because salt movement may control the thickness of reservoir formations. Figure 3 suggests that the fault system also controls the structurally lowest point of the Gassum reservoir (Fig. 5d). This could affect the storage capacity, which depends on the 3D geometry and permeability of the identified discontinuities and folds in the seismic data that define the reservoir and seal. Since follow-up 3D seismic investigations are essential, these surveys should determine whether the fault system extends continuously through the Jurassic-Cenozoic layers above the salt pillow. Additionally, they should assess potential leakage risks that could complicate lateral migration, such as reservoir compartmentalization^32^.

Geological storage of CO_2_ in the Thorning site is still possible, as the modelled reservoir and seal geometry are effective for storage of CO_2_ in the subsurface. While advantageous for storage, the structural high closure is more inclined to fractures and faulting, particularly those driven by the salt tectonics. The transecting faults may act as weakness zones and migration pathways that bypass impermeable seal formations and lead to CO_2_ escape from the reservoir to shallow groundwater systems and subsequently to the atmosphere. The migration of CO_2_ through the subsurface has the potential to affect the composition of the shallow groundwater aquifers. The presence of salt diapirs that intrude from the Zechstein Group into the Cenozoic succession south of the Thorning salt pillow, can induce faulting and be a source of shallow groundwater salinization. Salt movement was particularly active during the Cretaceous (Fig. 2) and persisted into the late Cenozoic, until the Miocene. An important question is whether it is still active and is worth detailed investigation for future GCS development work in the region. Since there are no seismicity records in the study area, it can be considered tectonically stable. However, this remains highly sensitive to potential changes as suggested by the persistence of salt movements until the Miocene. Furthermore, it is also important to consider recent studies for a possible interplay between ice-sheets and salt structures in sedimentary basins^33^, due to the presence of an ice-sheet margin in the study area.

The former ice margin of the last glaciation^34^, marked by the Main Stationary Line in Fig. 1b, where the palaeovalleys tend to terminate, explains the dense distribution of the palaeovalleys in the area. Drinking water aquifers may be present in the palaeovalleys, and the identified discontinuous and cross-cutting internal features of the valley infills suggest repeated episodes of erosion and subsequent deposition, which may influence groundwater dynamics. Considering that the palaeovalley incisions extend down into the Chalk Group, the hydrogeological network is likely interconnected with different aquifer systems. Therefore, palaeovalley mapping is critical for groundwater aquifer exploration and enhances our knowledge of groundwater vulnerability to GCS activities in regions affected by salt tectonics.

## Conclusions

Salt tectonics can significantly affect the saline aquifers targeted for carbon storage and the groundwater systems above. Using a dual-element seismic acquisition system comprising landstreamer and nodal recorders, we were able to image geological features at different depths with high-resolution. The new data reveal Quaternary palaeovalleys and faults that truncate the reservoirs and seals, which are associated with the salt pillow growth and diapirism. Notably, some of the faults appear continuous up to the base of the palaeovalleys. These findings form the basis for modelling the reservoir structure and fluid pathways, which is important for assessing the long-term safety and integrity of the carbon storage site.

## Methods

### Seismic data acquisition

The seismic data were collected during August–October 2023. Data were acquired along roads from 8 seismic profiles, 133 km long, specifically designed to cover the interpreted top Gassum closure^5^. The acquisition setup and parameters are shown in Supplementary Figure S6 and Supplementary Table S7, respectively. Two synchronized 12 tonne seismic sources were used for the seismic lines P1–P5, which were later replaced by a single 18 tonne source for the seismic lines P6–P8. The 18 tonne source was first tested and calibrated in the field to deliver an equivalent force to the two 12 tonne sources, ensuring that both setups are directly comparable. Three sweeps were generated every 10 m at the receiver location for a better signal-to-noise ratio. A linear 18 s long sweep with a 10–140 Hz bandwidth was chosen based on previous GCS surveys in Denmark during the scope of the same project. The shots were accurately time-stamped using a global positioning system (GPS) and simultaneously recorded by the nodal array and landstreamer. Fixed or asymmetric split-spread roll-along geometry of approximately 600–1200 live recorders with 10 m spacing was used for the array depending on the length of the profile. The position of the nodal recorders was measured using a differential global positioning system (DGPS). The landstreamer was towed by the rear seismic source along the side of the road adjacent to the nodal array. Depending on the conditions of the road, the landstreamer consists of 1 to 5 segments, each containing 20 three-component (3C) MEMS with 2 m spacing.

By using a combined nodal and landstreamer seismic acquisition setup, the salt pillow and suprasalt strata, including the palaeovalleys, were imaged with high-resolution using two complementary datasets. The nodal dataset offers good overall reflectivity but may require significant resources, such as densely spaced receivers, particularly in areas with complex geological structures and thin layers within unconsolidated shallow sediments. In contrast, the landstreamer provides a simple, efficient, and cost-effective solution.

Previous studies have used different types of landstreamers for buried valley mapping in Denmark^35^. In our study, the landstreamer is designed with 3C broadband MEMS system accelerometers. Compared with geophone nodal recorders, MEMS are less susceptible to electromagnetic noise from strong power lines in urban environments due to their fully digital transmission. The broadband response improves the vertical seismic resolution, allowing for the imaging of thinner seismic features based on the ratio between the maximum and minimum signal frequencies.

### Seismic data processing

The data processing flow is presented in Supplementary Table S8. The processing was performed with Globe Claritas™ V2023.2.0 seismic processing software [https://www.petrosys.com.au/claritas/]. The raw SEGD data were cross-correlated with the theoretical sweep, and the repeated shots were vertically stacked. Prestack processing included trace editing, geometry setup with 5 m common midpoint (CMP) spacing, first arrival picking, refraction static corrections, and minimum phase conversion using an empirically determined matching filter. The seismic data are displayed with the Society of Exploration Geophysicists (SEG) standard convention for a minimum phase wavelet, implying a downward increase (i.e., soft event) and a decrease (i.e., hard event) in acoustic impedance corresponding to a negative (white/red; trough) and positive (black/blue; peak) reflection^36^. The noise was targeted using a frequency wavenumber (FK) filter, time-dependent bandpass and airwave attenuation. Source-generated noise was removed with 50 Hz and 82 Hz notch filters. A predictive deconvolution filter was used to compress the wavelet and attenuate multiples. Surface-consistent residual static corrections and velocity analysis were repeated up to two times to account for severe static issues and improve seismic feature continuity. Examples of noise attenuation are shown in Supplementary Figures S9 and S10. The unmigrated stacked data were further filtered using a coherency filter (FX-deconvolution), amplitude balance, and bandpass filter. Migration was performed using a finite-difference migration algorithm.

While the landstreamer vertical component results that focus on primary wave reflection are sufficient for our current research, the simultaneously recorded radial and transverse component datasets hold the potential for higher-resolution shear wave imaging. Additionally, the raw data (Supplementary Figure S10) reveal reflectivity down to the top pre-Zechstein, which is imaged only in shot gathers with minimal groundroll noise. In cases where applicable, advanced noise attenuation solutions may be used for complementary deep reservoir imaging using the landstreamer dataset.

### Seismic data interpretation

We used lithostratigraphic data^21^ and publicly available reports from deep wells and legacy seismic profiles to place our seismic datasets within the stratigraphic framework of the sedimentary basin. These sources have collocated points from which seismic features could be traced to the study area, allowing for consistent geological interpretation. The seismic horizons and transecting faults were picked in all seismic profiles and used to create the structural time maps. The palaeovalleys were modelled using both the picked horizons and a palaeovalley map, which is based on prior electromagnetic surveys.

## Supplementary Information

Supplementary Information.

## Data Availability

The acquired seismic data can be accessed and downloaded through the https://www.geus.dk/produkter-ydelser-og-faciliteter/data-og-kort/ccs-data-2022-2024 of the Geological Survey of Denmark and Greenland (GEUS) and are archived at Uppsala University, by the reflection seismic group. All geophysical, well and log data are publicly available at https://eng.geus.dk/products-services-facilities/data-and-maps/subsurface-data-denmark and geological maps are publicly available at https://eng.geus.dk/products-services-facilities/data-and-maps/maps-of-denmark. The elevation model used to generate the map is publicly available at https://dataforsyningen.dk/.

## References

[1] Stille, H. The upthrust of the salt masses of Germany. *AAPG Bull.* **9**, 417–441. 10.1306/3D9326C3-16B1-11D7-8645000102C1865D (1925).

[2] Hudec, M. R. & Jackson, M. P. A. Terra infirma: Understanding salt tectonics. *Earth-Sci. Rev.* **82**, 1–28. 10.1016/j.earscirev.2007.01.001 (2007).

[3] Jackson, M. P. A. & Hudec, M. R. *Salt Tectonics: Principles and Practice* (Cambridge University Press, 2017).

[4] Trusheim, F. Mechanism of salt migration in Northern Germany. *AAPG Bull.* **44**, 1519–1540. 10.1306/0BDA61CA-16BD-11D7-8645000102C1865D (1960).

[5] Hjelm, L. et al. Capture, Storage and Use of CO2 (CCUS). Evaluation of the CO2 storage potential in Denmark. Vol.1: Report & Vol 2: Appendix A and B [Published as 2 separate volumes both with Series number 2020/46]. gpub 10.22008/gpub/34543 (2022).

[6] Hospers, J., Rathore, J. S., Jianhua, F., Finnstrøm, E. G. & Holthe, J. Salt tectonics in the Norwegian-Danish Basin. *Tectonophysics* **149**, 35–60. 10.1016/0040-1951(88)90117-5 (1988).

[7] Danielsen, J. E., Auken, E., Jørgensen, F., Søndergaard, V. & Sørensen, K. I. The application of the transient electromagnetic method in hydrogeophysical surveys. *J. Appl. Geophys.* **53**, 181–198. 10.1016/j.jappgeo.2003.08.004 (2003).

[8] Jørgensen, F., Sandersen, P. B. E. & Auken, E. Imaging buried Quaternary valleys using the transient electromagnetic method. *J. Appl. Geophys.* **53**, 199–213. 10.1016/j.jappgeo.2003.08.016 (2003).

[9] Jørgensen, F. & Sandersen, P. B. E. Buried and open tunnel valleys in Denmark-erosion beneath multiple ice sheets. *Quat. Sci. Rev.* **25**, 1339–1363. 10.1016/j.quascirev.2005.11.006 (2006).

[10] Jørgensen, L. F. & Stockmarr, J. Groundwater monitoring in Denmark: Characteristics, perspectives and comparison with other countries. *Hydrogeol. J.* **17**, 827–842. 10.1007/s10040-008-0398-7 (2009).

[11] Høyer, A. S., Jørgensen, F., Sandersen, P. B. E., Viezzoli, A. & Møller, I. 3D geological modelling of complex buried-valley network delineated from borehole and AEM data. *J. Appl. Geophys.* **122**, 94–102. 10.1016/j.jappgeo.2015.09.004 (2015).

[12] Sandersen, P. B. E. & Jørgensen, F. Kortlægning af begravede dale i Danmark. Opdatering 2010-2015. Bind 1: Hovedrapport (De Nationale Geologiske Undersøgelser for Danmark og Grønland, 2016).

[13] Papadopoulou, M. et al. Innovative land seismic investigations for CO2 geologic storage in Denmark. *Geophysics* **88**, 251–266. 10.1190/geo2022-0693.1 (2023).

[14] Bredesen, K., Smit, F., Lorentzen, M. & Gregersen, U. Improved delineation of the Gassum Formation reservoir zones using seismic impedance inversions: Implications for exploiting the Stenlille aquifer gas storage facility as a storage demonstration site, onshore Denmark. *Geoenergy* **1**, 1–12. 10.1144/geoenergy2022-002 (2023).

[15] Malehmir, A. et al. Fault intersections control short period intraplate start-stop seismicity in the Korean Peninsula. *Tectonophysics* **834**, 1–11. 10.1016/j.tecto.2022.229387 (2022).

[16] Zappalá, S. et al. Crustal-scale fault systems in the Korean peninsula unraveled by reflection seismic data. *Earth Space Sci.* **9**, 1–14. 10.1029/2022EA002464 (2022).

[17] Zappalá, S. et al. Combined onshore and offshore wide-scale seismic data acquisition and imaging for carbon capture and storage exploration in Havnsø, Denmark. *Geophysics* **89**, 257–272. 10.1190/geo2023-0503.1 (2024).

[18] Malehmir, A., Markovic, M., Abramovitz, T. & Gregersen, U. Geological carbon storage site characterization using a dual element seismic recording technology. *Sci. Rep.* **15**, 1–13. 10.1038/s41598-025-96012-8 (2025). 40234639 10.1038/s41598-025-96012-8 PMC12000295

[19] Westgate, M. et al. Seismic imaging of halokinetic sequences and structures with high-resolution, dual-element acquisition, and processing: Applications to the Gassum structure in eastern Jutland, Denmark. *Earth Space Sci.* **1–15**, 2025. 10.1029/2024EA004014 (2025).

[20] Vejbæk, O. V. & Britze, P. Geologisk kort over Danmark. Geological map of Denmark 1:750 000. Top præ-Zechstein (to vejs løbetid og dybde). Top pre-Zechstein (two-way traveltime and depth). DGU Kortserie **45**, 1–9. 10.22008/FK2/F9DWMB/IMCMD5 (1994).

[21] Nielsen, L. H. & Japsen, P. Deep wells in Denmark 1935-1990. Lithostratigraphic subdivision (Danmarks Geologiske Undersøgelse Serie A, 1991).

[22] Madirazza, I. & Jacobsen, B. H. Nøvling: An unusual salt structure on the southern margin of the Danish Zechstein Basin. *Bull. Geol. Soc. Denmark*. **44**, 139–149. 10.37570/bgsd-1998-44-08 (1998).

[23] Nielsen, L. H. Late Triassic–Jurassic development of the Danish Basin and the Fennoscandian Border Zone, southern Scandinavia. *GEUS Bull*. **1**, 459–526. 10.34194/geusb.v1.4681 (2003).

[24] Andersen, H. L. & Surlyk, F. The Cretaceous-Palaeogene boundary at Stevns Klint, Denmark: Inversion tectonics or sea-floor topography?. *J. Geol. Society* **161**, 343–352. 10.1144/0016-764903-021 (2004).

[25] Bjerager, M. & Surlyk, F. Benthic palaeoecology of Danian deep-shelf bryozoan mounds in the Danish Basin. *Palaeogeogr. Palaeoclimatol. Palaeoecol.* **250**, 184–215. 10.1016/j.palaeo.2007.03.008 (2007).

[26] Bjerager, M., Surlyk, F., Andersen, H. L., Thibault, N. & Stemmerik, L. Danian cool-water coral reefs in southern Scandinavia localised over seafloor highs. *Mar. Petroleum Geol.* **27**, 455–466. 10.1016/j.marpetgeo.2009.09.008 (2010).

[27] Smed, P. Die Entstehung der dänischen und norddeutschen Rinnentäler (Tunneltäler) - Glaziologische Gesichtspunkte. E &G - Quaternary Science Journal **48**, 1–18. 10.23689/fidgeo-1398 (1998).

[28] Boldreel, L. O. On the structural development of the salt dome province in NW Jutland, Denmark, based on seismic studies. *First Break* **3**, 1–7. 10.3997/1365-2397.1985015 (1985).

[29] Geil, K. The development of salt structures in Denmark and adjacent areas: the role of basin floor dip and differential pressure. *First Break* **9**, 1–17. 10.3997/1365-2397.1991022 (1991).

[30] Frederiksen, S., Nielsen, S. B. & Balling, N. A numerical dynamic model for the Norwegian-Danish Basin. *Tectonophysics* **343**, 165–183. 10.1016/S0040-1951(01)00223-2 (2001).

[31] Andsbjerg, J., Nielsen, L. H., Johannessen, P. N. & Dybkjær, K. Divergent development of two neighbouring basins following the jurassic north sea doming event: The Danish central graben and the Norwegian-Danish Basin. *Norwegian Petroleum Society Special Publ.* **10**, 175–197. 10.1016/S0928-8937(01)80013-8 (2001).

[32] Jolley, S. J., Fisher, Q. J., Ainsworth, R. B., Vrolijk, P. J. & Delisle, S. Reservoir Compartmentalization (Geological Society of London, 2010).

[33] Hardt, J., Dooley, T. P. & Hudec, M. R. Physical modeling of ice-sheet-induced salt movements using the example of northern Germany. *Earth Surface Dynamics* **12**, 559–579. 10.5194/esurf-12-559-2024 (2024).

[34] Nielsen, M. H. Signature and timing of the Kattegat Ice Stream: Onset of the Last Glacial Maximum sequence at the southwestern margin of the Scandinavian Ice Sheet. *Boreas* **32**, 227–241. 10.1111/j.1502-3885.2003.tb01439.x (2008).

[35] Pedersen, T. V., Rasmussen, E. S. & Kristensen, M. Detailed mapping of Miocene sand-rich deposits in Denmark with high-resolution 2D land streamer vibroseis. *First Break* **30**, 45–50. 10.3997/1365-2397.30.8.60904 (2012).

[36] Sheriff, R. E. Encyclopedic Dictionary of Applied Geophysics (Society of Exploration Geophysicists, 2002).

