# Supplementary material for: The role of a salt pillow in deep saline aquifer integrity and shallow groundwater resources
Source: Sci Rep. 2025 Apr 29;15:15074. doi: 10.1038/s41598-025-99721-2 (PMC12041373; doi:10.1038/s41598-025-99721-2)
Supplement: Supplementary file 1 — Supplementary Information. [file 41598_2025_99721_MOESM1_ESM.pdf]

The role of a salt pillow in deep saline aquifer integrity and shallow groundwater resources

Jolanta Putnaite[1,*], Alireza Malehmir[1], Morten Bjerager[2], Tanni Abramovitz[2], Henrik Vosgerau[2], and Marie Keiding[2]

[1]Uppsala University, Department of Earth Sciences, Uppsala, SE-75236, Sweden
[2]Geological survey of Denmark and Greenland, Copenhagen, DK-1350, Denmark
[*]jolanta.putnaite@geo.uu.se

Supplementary Figures

Supplementary Figure S1.

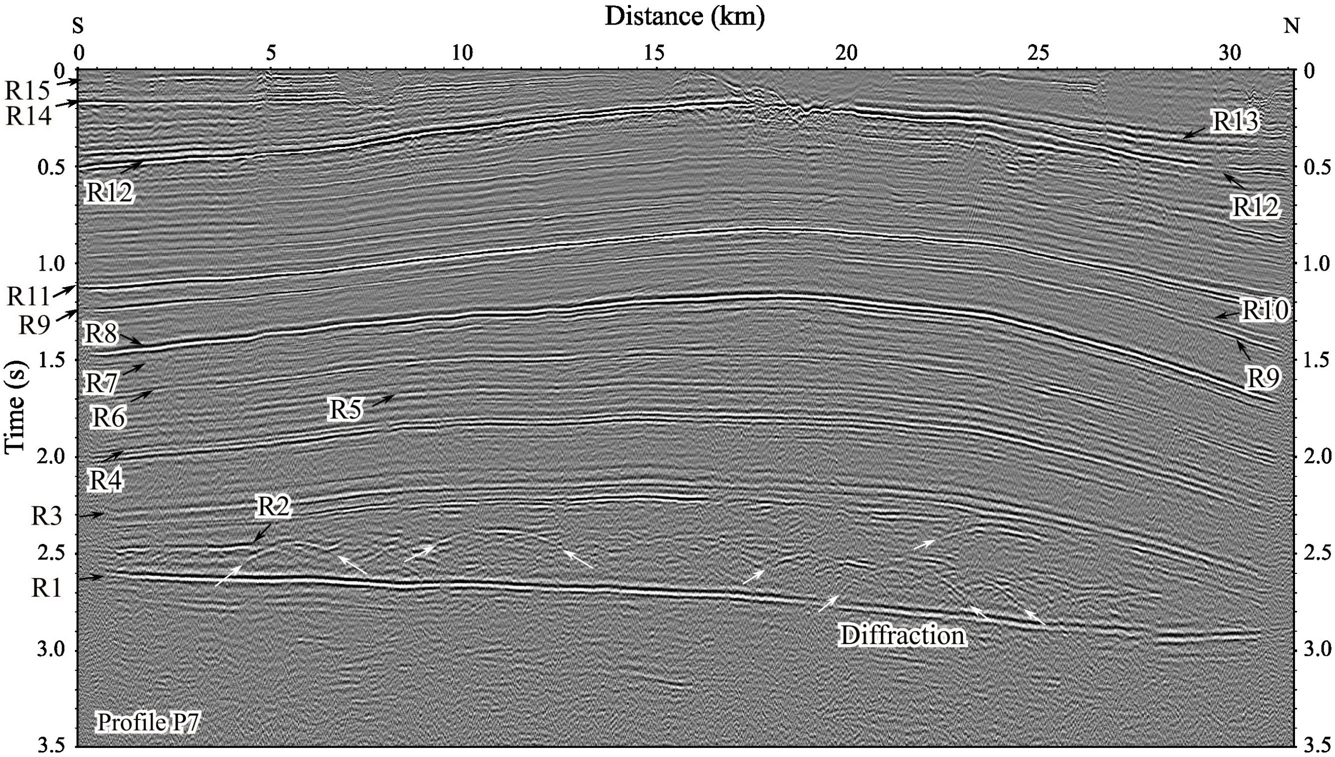

Figure S1. Example of an unmigrated stacked section of the nodal data from P7 traversing the Thorning salt pillow. The primary seismic features are R1–R15. The white arrows indicate diffractivity within the salt pillow. Figure was made using SKUA-GOCAD22 [https://www.aspentech.com/en/products/sse/aspen-skua] and Inkscape 1.3.2 [https://inkscape.org/].

Supplementary Figure S2.

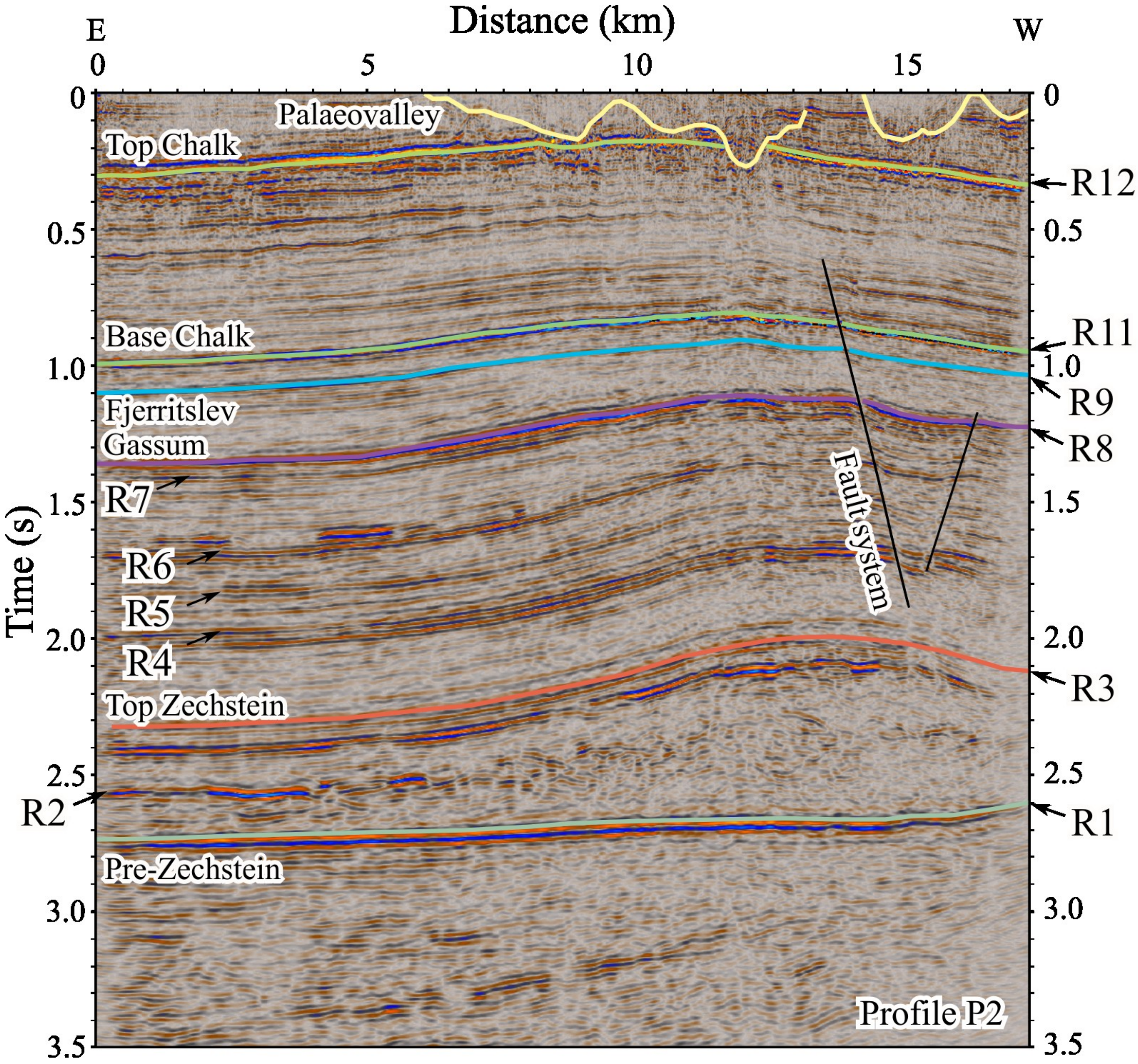

Figure S2. **Migrated stacked section of the nodal data from P2 running through the centre of the Thorning salt pillow**. The primary seismic features and the palaeovalley boundaries are traced across the section and shown using the corresponding colours. Black lines mark the fault system. Figure was made using SKUA-GOCAD22 [https://www.aspentech.com/en/products/sse/aspen-skua] and Inkscape 1.3.2 [https://inkscape.org/].

Supplementary Figure S3.

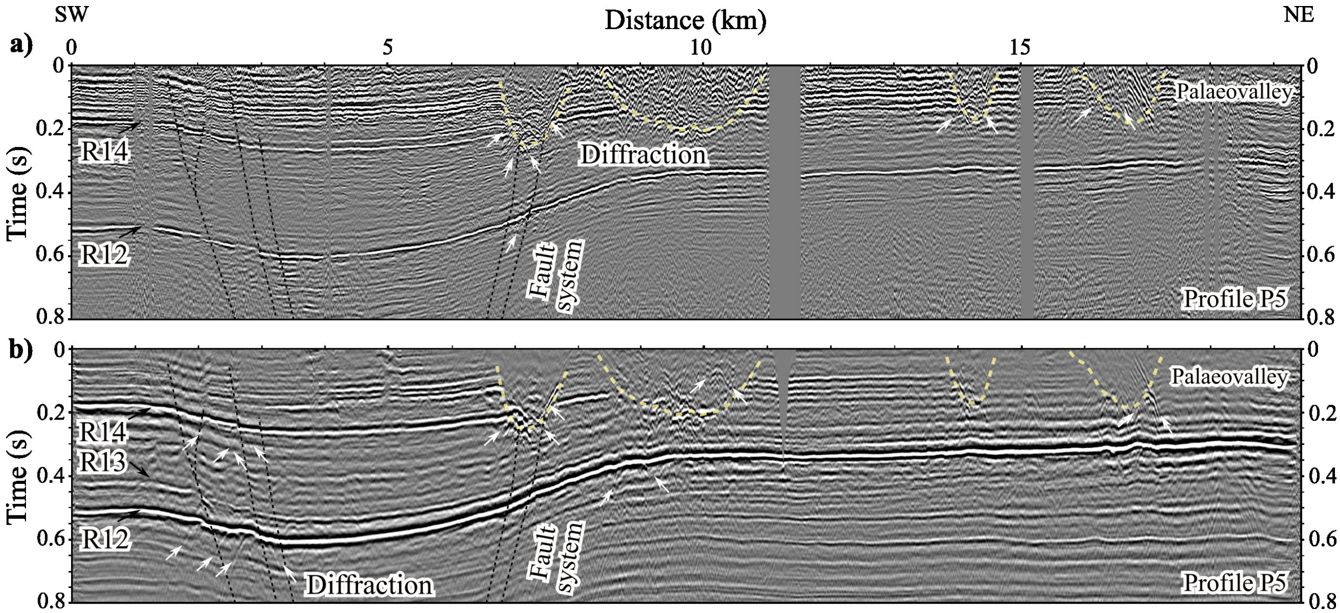

Figure S3. Near-surface portion of the unmigrated stacked section of the nodal seismic data from P5 traversing the fault system. **(a)** The landstreamer data and **(b)** the nodal data. The palaeovalley boundaries are marked by yellow dashed lines. Black discontinuous lines mark the fault system and the white arrows indicate diffractivity. Figures were made using SKUA-GOCAD22 [https://www.aspentech.com/en/products/sse/aspen-skua] and Inkscape 1.3.2 [https://inkscape.org/].

Supplementary Figure S4.

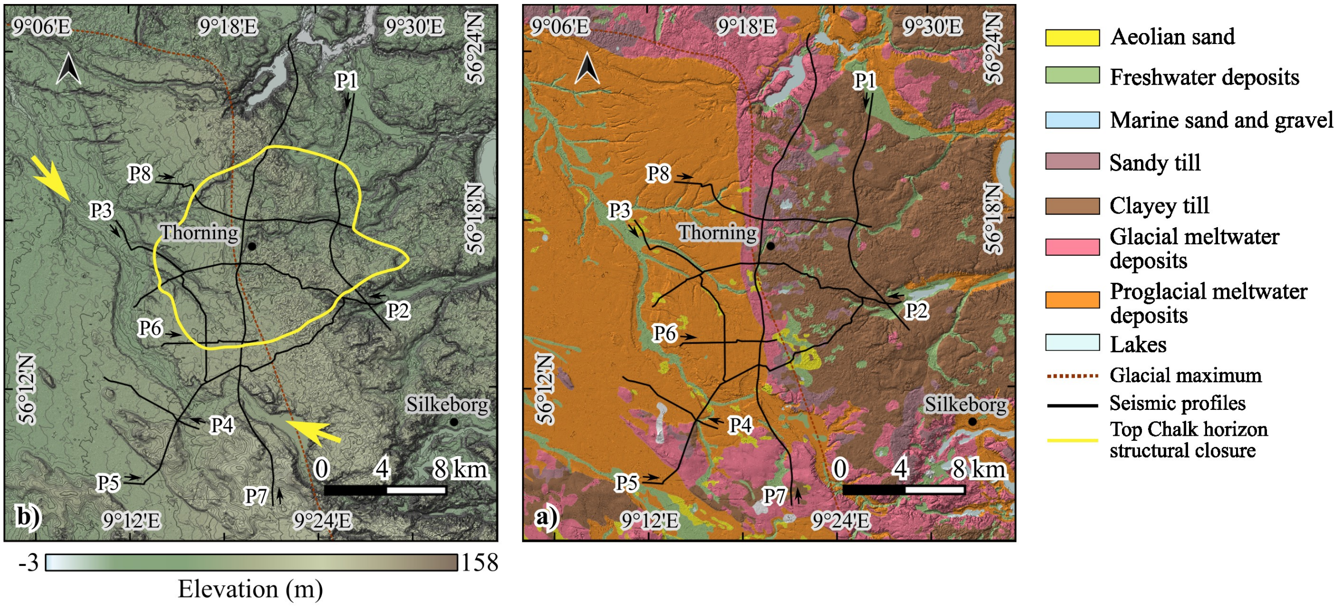

Figure S4. Elevation and Quaternary geology maps of the study area. **(a)** Detailed height model showing the palaeoriver valley expression on the surface. The contour lines are drawn at 2 m intervals, with bold lines marking every 10 m. The yellow polygon indicates the top Chalk horizon structural closure while the yellow arrows point out the palaeoriver valley running along the SW edge of the structure. **(b)** Surface geological map of the glacial landscape in the east and the proglacial meltwater plain in the west. Maps were made using QGIS 3.22 [https://www.qgis.org/] and underlying data were extracted from open data sources [https://eng.geus.dk/products-services-facilities/data-and-maps/maps-of-denmark; https://dataforsyningen.dk/data/3931].

Supplementary Figure S5

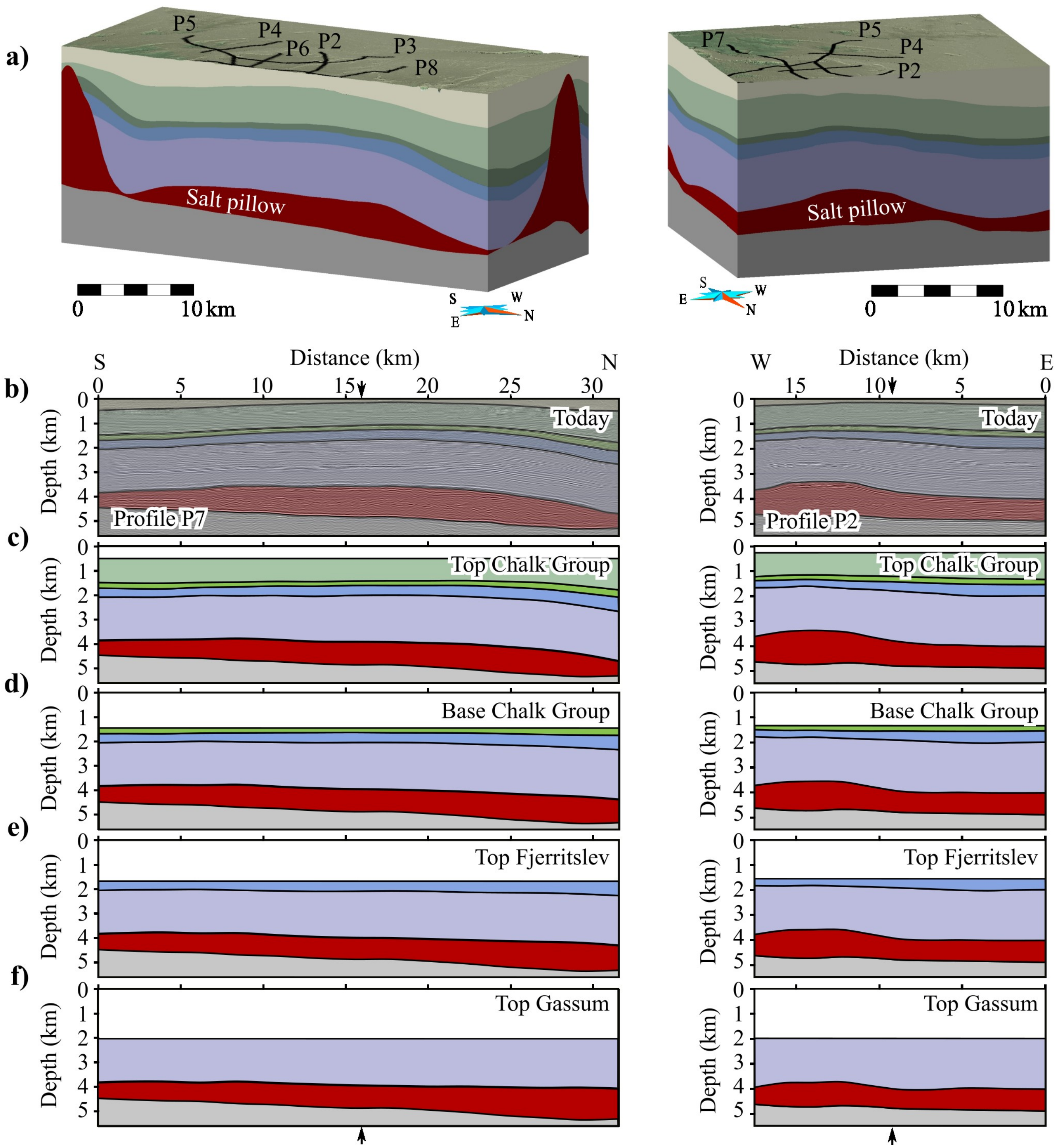

Figure S5. Geological model of the Thorning site. **(a)** 3D view of the geological model along P7 (left) and P2 (right). An ideal 2D representation of salt tectonics through time for seismic sections P7 and P2: **(b)** present, **(c)** Danian, **(d)** Lower Cretaceous, **(e)** Jurassic, **(f)** Upper Triassic. The black arrows indicate locations where the profiles intersect. Figures were made using Inkscape 1.3.2 [https://inkscape.org/].

Supplementary Figure S6

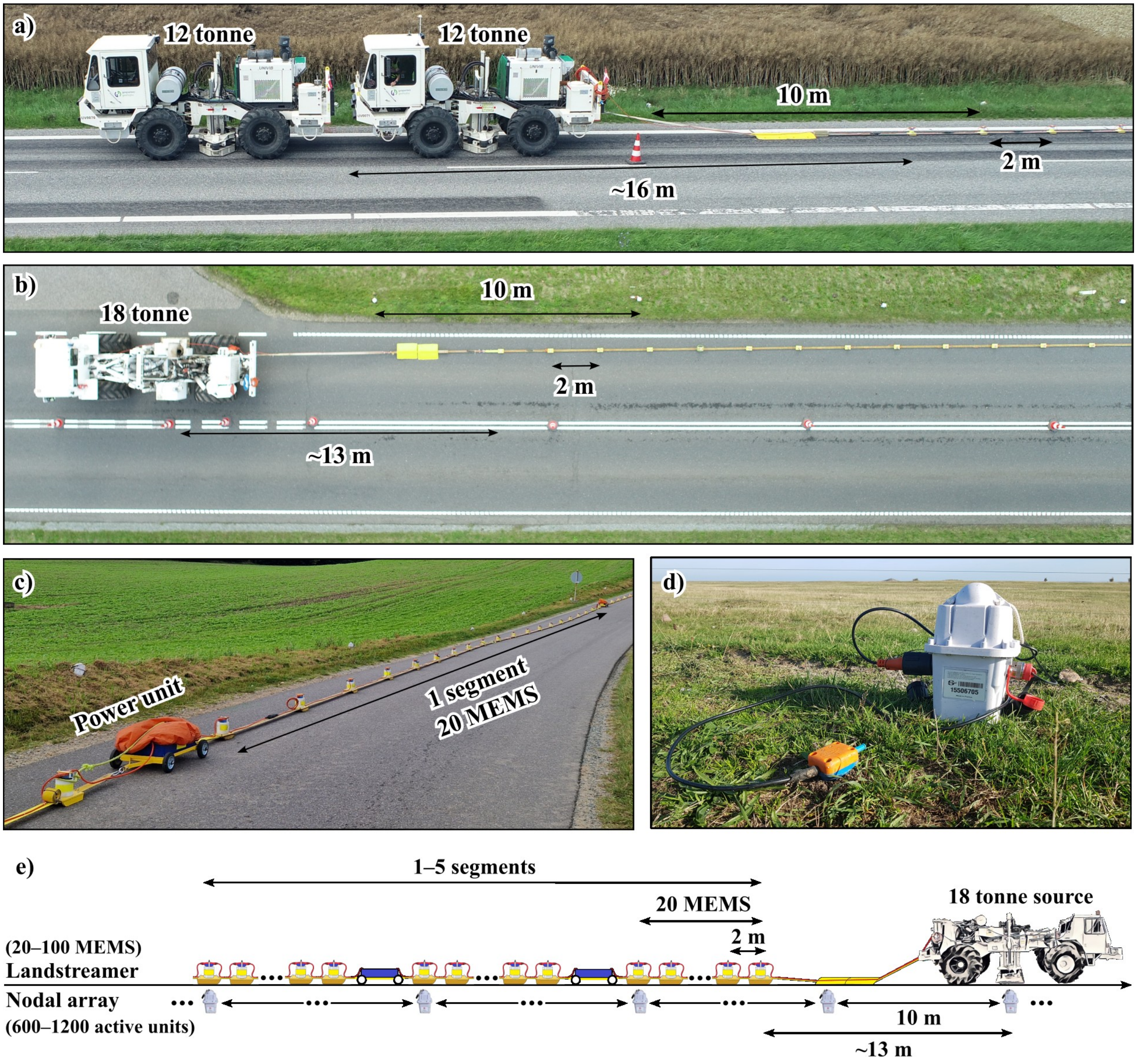

Figure S6. Seismic data acquisition setup. Drone perspective of **(a)** two 12 tonne seismic source solution and **(b)** one 18 tonne seismic source solution. Example of **(c)** a landstreamer segment and **(d)** a nodal unit connected to a geophone. **(e)** Schematic representation of the acquisition system. Figures were made using Inkscape 1.3.2 [https://inkscape.org/].

Supplementary Table S7

Table S7. Key elements of the seismic data acquisition, Thorning area (August–October, 2023).

Acquisition parameters	
Geodetic surveying	Reach RX RTK DGPS
Geometry	Fixed or asymmetric split-spread, roll-along
Source type	INOVA UNIVIB-326 (2 x 12 tonne, peak force of 95 kN per truck; profiles 1–5, 7)
	BIRDWAGEN MARK IV (1 x 18 tonne, peak force 128 kN; profiles 6–8)
Source sweep	10-140 Hz linear, 18 s long, 3 sweeps per shot point
Recording system	Sercel lite™
Landstreamer sensors	MEMS 3C, 1 ms sampling interval
Nodal array receivers	10 Hz spike, 2 ms sampling interval
Shot spacing	10 m
MEMS spacing	2 m
Nodal receivers spacing	10 m
Record length	25 s

Supplementary Table S8

Table S8. Key processing steps of the landstreamer and nodal datasets.

Nr.	Processing step	Landstreamer	Nodal
1.	Import SEGD data	✓	✓
2.	Cross-correlation with theoretical sweep	✓	✓
3.	Vertical stack of repeaded shots (diversity)	✓	✓
4.	Trace editing	✓	✓
5.	Geometry setup and CMP binning	5 m CMP spacing	
6.	Conversion to minimum phase	✓	✓
7.	First arrival picking		✓
8.	Elevation statics	Topographic statics using replacement velocity of 1700 m/s and reference elevation of 130 m	
9.	Refraction statics	✓	✓
10.	FK filter		✓
11.	Surface wave denoising		✓
12.	Gapped deconvolution	18 ms	24 ms
13.	Bandpass filter	20–30–135–140 Hz	10–20–120–140 Hz
14.	Amplitude balance	AGC 300	Rolling balance
15.	Airwave attenuation		✓
16.	Notch	48–52 Hz, 80–84 Hz	
17.	Velocity analysis		✓
18.	Reflection-based residual statics	Nodal residual statics + one round	Two rounds
19	NMO corrections	Picked stretch mute	
20.	Stack	✓	✓
21.	FX-deconvolution coherency filter	✓	✓
22.	Amplitude balance	Rolling balance	
23.	Bandpass filter	20–40–130–135 Hz at 0–720 ms; 20–30–130–135 Hz at 770–5000 ms	15–25–120–140 Hz at 0–720 ms; 10–20–130–140 Hz at 770–5000 ms
24.	Seismic reference datum correction	✓	✓
25.	Mean sea-level correction	✓	✓
26.	Finite difference post-stack migration	✓	✓

Supplementary Figure S9

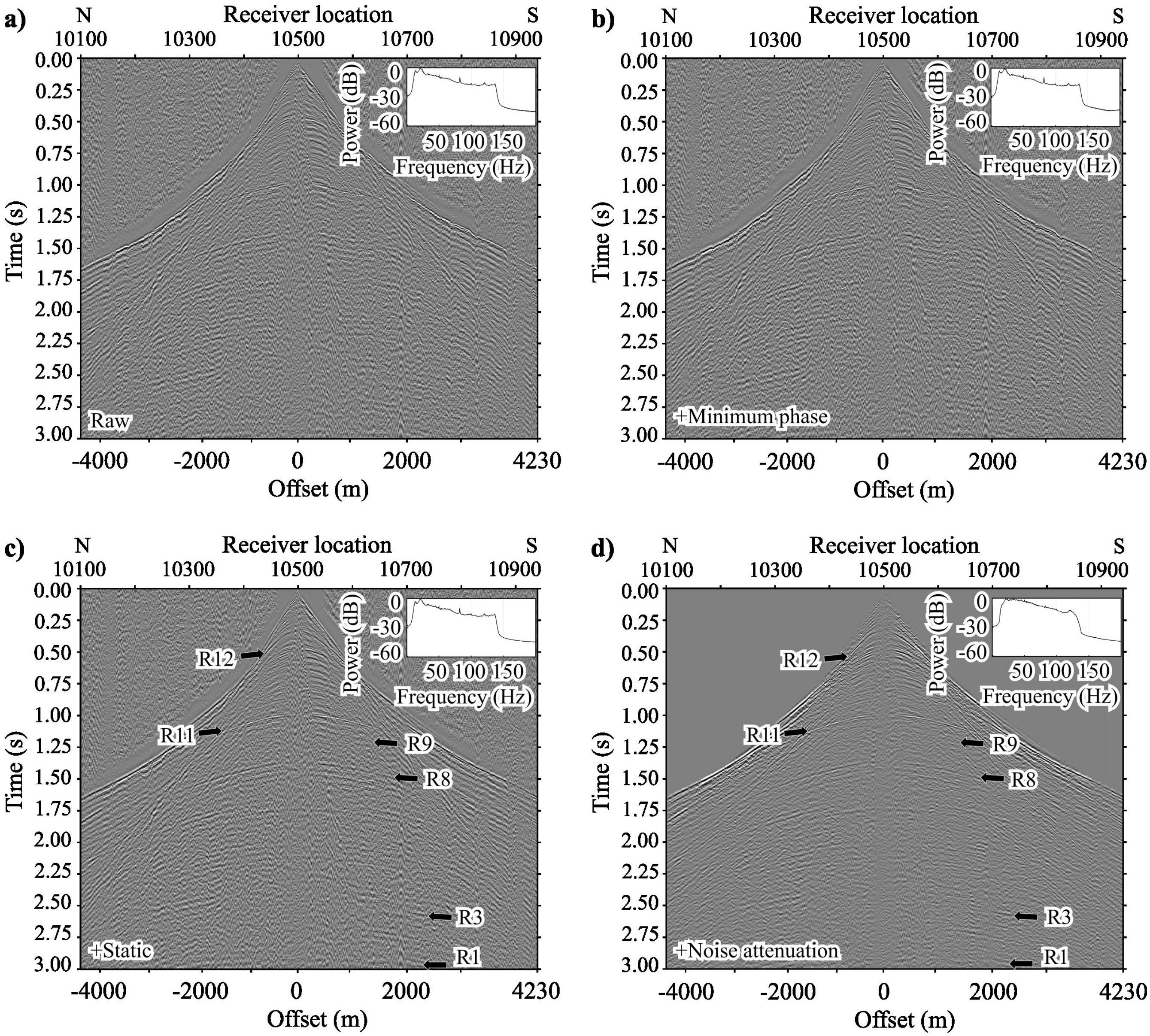

Figure S9. Example of a shot gather from the nodal dataset showing the effect of different pre-stack processing steps. (**a**) Raw data, (**b**) after minimum phase conversion, (**c**) after elevation and refraction static corrections, and (**d**) after noise attenuation. Figures were made using Inkscape 1.3.2 [https://inkscape.org/].

Supplementary Figure S10

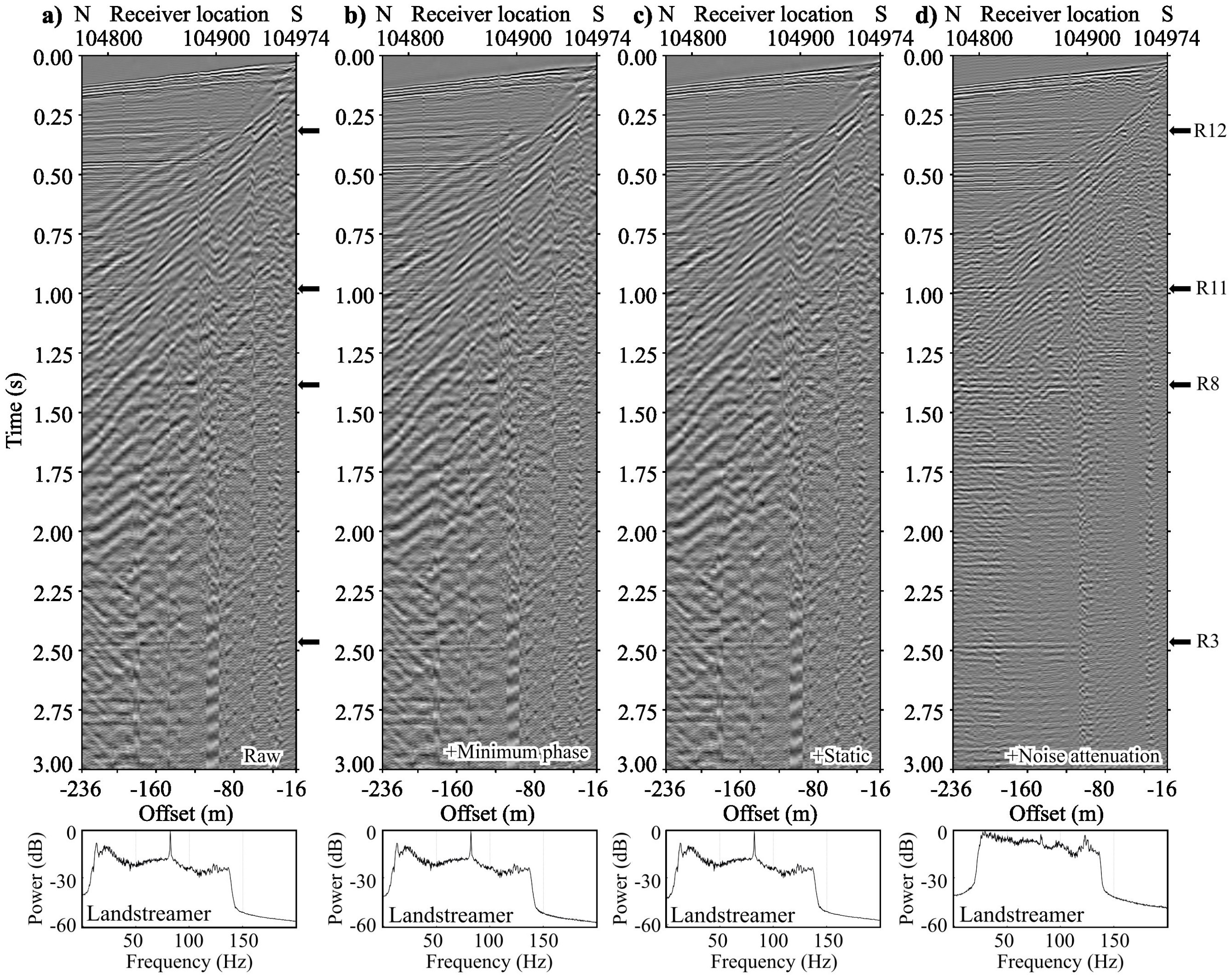

Figure S10. **Example of a shot gather from the landstreamer dataset showing the effect of various pre-stack processing steps**. (**a**) Raw data, (**b**) after minimum phase conversion, (**c**) after elevation and refraction static corrections, and (**d**) after noise attenuation. Figures were made using Inkscape 1.3.2 [https://inkscape.org/].
